# Prevention of Adhesions after Bone Fracture Using a Carboxymethylcellulose and Polyethylene Oxide Composite Gel in Dogs

**DOI:** 10.3390/vetsci11080343

**Published:** 2024-07-29

**Authors:** Aikaterini I. Sideri, Elena I. Pappa, Vassilis Skampardonis, Mariana Barbagianni, Stefanos G. Georgiou, Dimitra Psalla, Christina Marouda, Nikitas N. Prassinos, Apostolos D. Galatos, Pagona G. Gouletsou

**Affiliations:** 1 Clinic of Surgery, School of Health Sciences, Faculty of Veterinary Science, University of Thessaly, Trikalon 224, GR 43100 Karditsa, Greece; ksideri@uth.gr (A.I.S.); elenpapp@uth.gr (E.I.P.); mabarbag@uth.gr (M.B.); stegeorgiou@uth.gr (S.G.G.); agalatos@vet.uth.gr (A.D.G.); 2 Laboratory of Epidemiology, Biostatistics and Animal Health Economics, School of Health Sciences, University of Thessaly, Trikalon 224, GR 43100 Karditsa, Greece; bskamp@uth.gr; 3 School of Veterinary Medicine, Aristotle University of Thessaloniki, Stavrou Voutira 11, GR 54124 Thessaloniki, Greece; dpsalla@auth.gr (D.P.); cmarouda@auth.gr (C.M.); ngreen@auth.gr (N.N.P.); 4 Clinic of Obstetrics and Reproduction, Faculty of Veterinary Science, School of Health Sciences, University of Thessaly, Trikalon 224, GR 43100 Karditsa, Greece

**Keywords:** adhesion, anti-adhesion barrier, bone fracture, carboxymethylcellulose, dog, gel, polyethylene oxide

## Abstract

**Simple Summary:**

This study evaluated the use of a carboxymethylcellulose (CMC)/polyethylene oxide (PEO) composite material to prevent adhesions following orthopedic surgery in dogs. Ten purpose-bred laboratory Beagle dogs were allocated consecutively into two groups—one received the anti-adhesion material during an ulna osteotomy, while the other group did not. The results showed no significant differences between the groups in terms of surgical site size, lameness, or bone healing on imaging evaluation. The group that received the anti-adhesion material had significantly lower adhesion and better healing scores based on macroscopic and histopathologic evaluation. These findings suggest the CMC/PEO composite is a safe and potentially effective way to prevent post-surgical adhesions in canine orthopedic patients, without compromising bone healing. Further research is needed to fully characterize the clinical benefits of this approach.

**Abstract:**

The formation of adhesions is a common complication following traumatic injuries and surgical procedures, often resulting in pain, stiffness, and loss of function. This study aimed to evaluate the feasibility and safety of using a composite material comprising of carboxymethylcellulose (CMC), polyethylene oxide (PEO), and calcium chloride, for preventing adhesions between muscle and bone during the healing stage, as well as its effect on the bone healing process. Ten healthy purpose-bred laboratory Beagle dogs were randomly subjected to two consecutive operations with a 6-month interval, alternating between left and right forelimbs. On the left forelimb an osteotomy at the ulna was performed, while on the right forelimb the same procedure was supplemented by the application of the anti-adhesion agent in the osteotomy site prior to closure. Clinical, diagnostic imaging, macroscopic, and histological evaluations were performed at various time points. The results showed no significant differences in surgical site perimeter (*p* = 0.558), lameness (*p* = 0.227), and radiographic bone healing (*p* = 0.379) between the two groups. However, the macroscopic (*p* = 0.006) and histological assessments revealed significantly lower adhesion scores (*p* = 0.0049) and better healing (*p* = 0.0102) in the group that received the anti-adhesion agent. These findings suggest that the CMC/PEO composite material is a safe and potentially effective intervention for preventing post-traumatic and post-surgical adhesions in canine patients without compromising bone healing. Further research is warranted to fully characterize the clinical benefits of this approach.

## 1. Introduction

The formation of adhesions represents one of the most common causes of complications during the post-traumatic period in all body tissues. Adhesions between tissues occur as a result of tissue trauma, reaction to a foreign body, bleeding, or a microbial agent [1,2]. They start to form during the early stages of wound healing, between days 2 and 7 post-injury, as part of the normal physiological process involving inflammation and coagulation [2,3]. During this period, they are considered reversible, as the presence of collagen in the area is not yet extensive. Even though the mechanism of adhesion formation is not fully understood, it is known that their formation is driven by an increase in fibrin deposition, ongoing inflammation, and reduced fibrin breakdown. This means that, if the fibrin clots are not effectively dissolved, they provide a framework that, along with new blood vessel growth, leads to the formation of adhesions [1,4,5].

In the post-operative period, the formation of adhesions between organs and tissues is often irreversible and constitutes a major factor of post-operative complications. The severity of them and their clinical signs can differ depending on the specific tissue they are located in. In orthopedic surgery, adhesions are often responsible for pain, stiffness, and loss of function in the affected limbs [4]. Peritendinous adhesions are widely investigated, as they lead to a loss of the gliding properties and overall function of the tendon, thereby limiting the range of motion [2,3,4,5,6]. Joint fibrosis is characterized by the formation of adhesions, which often lead to joint stiffness and subsequently increase the risk of cartilage [7,8,9]. In small animals, fractures of the femur are frequently accompanied by substantial trauma of the quadriceps femoris muscle. This can lead to the development of adhesions between the muscle, the femur, and the surrounding tissues. This adhesion formation often results in severe and typically irreversible post-operative complications, particularly in young patients, a condition referred to as quadriceps muscle contracture [9,10].

The principle of “prevention is preferable to treatment” is particularly applicable when it comes to the avoidance of adhesion formation post-operatively. In this regard, numerous strategies have been proposed, most of them concerning surgical conditions [1,4,9,11,12,13], but also the use of pharmaceutical agents [1,4,11], and post-operative controlled physical activity and physiotherapy [4,9]. The use of biodegradable materials that act as barriers between traumatic surfaces has emerged recently as a particularly useful tool in human medicine, aiming to prevent the formation of adhesions during the initial stages of healing [1,6,14] Many different materials, such as membranes, gels, and liquids, both biological and synthetic, have been tested. Ideally, a barrier should not actively interfere with inflammation and wound-healing processes but should separate the injured surfaces and allow them to heal without forming any adhesions. To achieve this, such barriers should be biocompatible and slowly degradable [12].

Antiadhesive barriers (membranes or gel barriers) consisting of materials of biological origin and their derivatives, synthetic polymers, or combinations have been tested in numerous studies in laboratory animals as well as in human clinical practice with very positive results [14,15]. In orthopedic surgery, the study and application of these materials are mainly focused on preventing the formation of post-operative adhesions in procedures aimed to repair wounds or tendon deficiencies [15,16,17,18,19,20]. Commonly investigated anti-adhesive barriers include hyaluronic acid and cellulose derivative products. Most of these studies have demonstrated promising results regarding their safety, easiness of application, and relative efficacy of these products [3,17,18,20]. A gel-based anti-adhesive material, comprising carboxymethylcellulose (CMC), polyethylene oxide (PEO), with calcium chloride and sodium chloride in water (Oxiplex; FzioMed, Inc., San Luis Obispo, CA, USA), has been shown in several studies to be safe, easy to use, and effective in reducing the formation of adhesions [21,22,23,24,25].

To the best of our knowledge, there is a paucity of information regarding the use of anti-adhesive barriers in the context of traumatic musculoskeletal injuries in canine patients.

Therefore, the objectives of the present study were threefold: (a) to evaluate the feasibility and safety of utilizing the composite material CMC/PEO for the prevention of adhesions between muscle and bone during the healing stage; (b) to assess the effect of the aforementioned material on the process of bone healing; and (c) to investigate the potential of this intervention to decrease the incidence of adhesions following orthopedic surgery.

## 2. Materials and Methods

### 2.1. Ethics: Animals

The study was conducted at the research facility area of the Clinic of Surgery, Faculty of Veterinary Science, University of Thessaly, Karditsa, Greece, and had been approved by the Greek National Animal Ethics Committee (license number 127440/22.6.2020), which confirmed that the study complied with the standards of the national and EU legislation regarding animal experimentation.

A total of 10 healthy purpose-bred laboratory Beagle dogs (8 males, 2 females) aged between 3 and 8 years were included in this study. Before, during, and after the experimental period, the animals were housed individually in spacious pens with access to outdoor areas and ample space for rest, at the experimental facilities of the Clinic. The animals received a standard commercial dry food diet and had unlimited access to water. Physical examination, complete blood count, and serum biochemical analyses of the animals were within normal limits; furthermore, the animals were routinely treated for parasites and vaccinated according to schedule. The study was conducted in alignment with EU regulations and ensured appropriate pain management and handling procedures were followed throughout the entire study period.

### 2.2. Overall Study Design

The animals underwent two consecutive operations, one on the left (control group) and one on the right forelimb (treatment group). The sequential procedures performed on the same dog were carried out at an interval of 6 months to minimize bias. In the control group (group A), the animals underwent only an osteotomy at the middle third of the ulna diaphysis of the left forelimb, followed by soft tissue closure; for the animals in the treatment group (group B), the CMC/PEO gel was applied in the interspace of the osteotomy site and the ulnar muscles of the contralateral limb before skin closure. Clinical examination and diagnostic imaging were performed the day before surgery (D − 1). In the post-operative period, these procedures were conducted at specific time intervals, as described below. In D28 all animals underwent a second surgery, and a sample was taken from the site of interest for histological evaluation.

### 2.3. Surgical Procedure

The animals were deprived of food overnight and had free access to water up to 2 h before the induction of anesthesia. Premedication included the administration of 0.05 mg/kg acepromazine (Acepromazine, Alfasan, Woerden, The Netherlands) and 0.1 mg/kg morphine (Morfina Cloridrato, Molteni, Scandicci, Italy) intramuscularly (im). Approximately 30 min later, anesthesia was induced with intravenous (iv) propofol (Propofol, Fresenius Kabi, Athens, Greece) increments until tracheal intubation and maintained with a mixture of isoflurane (Isoflo, Abbott, Maidenhead, Berkshire, UK) in oxygen.

As soon as anesthesia was induced, cefuroxime sodium (Zinacef, GlaxoSmithKline, Athens, Greece) was administered at a dose rate of 20 mg/kg, iv. The animals were positioned in lateral recumbency with the left (group A) or right (group B) forelimb positioned uppermost. The surgical area, distal to the elbow joint, was aseptically prepared. A skin incision was centered over the lateral edge of the ulna, starting from the proximal third and extending to the styloid process of the bone. The subcutaneous fat and superficial antebrachial fascia were incised on the same line, between the tendons of the ulnaris lateralis and the lateral digital extensor muscle. Retraction of the tendons and fascia exposed the middle third of the ulna diaphysis. At this point, a periosteal elevation measuring 2 cm in length and 0.5 cm in width was performed on the lateral surface of the ulna in order to create a traumatic surface on the bone. In the center of the aforementioned area, a complete cross-section osteotomy under continuous saline irrigation, using an oscillating bone saw (Veterinary Instrumentation, Sheffield, UK), was performed. A traumatic surface of a corresponding extent was also created in the ulnar muscle by removing a section of the epimysium. The entire surgical area was meticulously washed with normal saline to remove any clots. In the right forelimb (group B), one layer of CMC/PEO gel (Oxiplex; FzioMed, Inc., San Luis Obispo, CA, USA) was applied in the space between the osteotomy area and the adjacent muscles (Figure S1). In both groups, the antebrachial fascia, the subcutaneous, and the skin layers were closed in a routine suturing pattern.

Post-operative analgesia was provided by subcutaneous (sc) administration of meloxicam (Metacam, Boehringer Ingelheim, Ingelheim, Germany) 0.1 mg/kg, immediately after the end of the surgical procedure and every 24 h thereafter for 5 consecutive days. Furthermore, morphine 0.1 mg/kg was administered every 6 h for the first 24 h, and gabapentin (Neurontin, Pfizer Hellas, Athens, Greece) 10 mg/kg was administered orally every 8 h for 30 days. Additional pharmaceutical treatment included the administration of amoxicillin/clavulanic acid (Synulox, Haupt Pharma, Latina, Italy) orally, at a dose rate of 20 mg/kg, twice daily, until the 15th post-operative day.

Post-operatively, the animals were allowed to move freely as tolerated.

### 2.4. Post-Operatively Evaluation

#### 2.4.1. Clinical Evaluation

Daily visual inspection of the surgical wound was performed, and the perimeter of the site was measured weekly until D28. Additionally, the animal’s lameness was evaluated daily until D7, and then weekly until D28, with a score from 0 (absence of lameness) to 5 (non-weight-bearing) assigned based on a standardized assessment system [26] (Table S1).

#### 2.4.2. Diagnostic Imaging

Ultrasonographic scans were performed by the same experienced radiologist (M.B.) on days 0, 5, 10, 20, and 28 of the experimental period. The examination involved longitudinal scans using ultrasonographic equipment (MyLab^®^ 30; ESAOTE SpA, Genova, Italy) equipped with a linear transducer. The examinations evaluated the stage of bone healing by assessing the echogenicity and structural organization of the tissue at the fracture site, as well as by examining the formation of the callus and the vascularization present at the osteotomy (surgical bone-cutting) site. The work was performed with the animal in lateral recumbency after hair removal of the antebrachium and coupling gel was applied. The scans were performed along the dorsal surface of the ulna, imaging the bone from the proximal to the distal, including the osteotomy area. The following settings were used for the assessment: frequency of 15–18 MHz, imaging depth of 3 to 5 cm, and gain of 52–76%. The color Doppler settings used for vascularization assessment were kept the same as B-mode and pulse repetition frequency was 1.4 KHz. The findings were then scored from 1 (worst) to 5 (best) at a modified evaluation scale [27] (Table S2). On each examination, three images were taken and each of them was scored separately. The median value of the three scores was then considered as the final assessment.

Post-operatively, standard digital craniocaudal and mediolateral X-ray images were obtained on D1 and then on D28. The findings on D1 and D28, related to bone formation, union, and remodeling were scored using the modified Lane and Sandhu scoring system, which ranges from 0 (worst) to 10 (best) [28] (Table S3).

#### 2.4.3. Macroscopic and Histological Evaluation

On D28, the animals underwent a subsequent surgical procedure at the same site. The extent and ease of separating any adhesions were macroscopically examined. The findings were evaluated by two surgeons (A.I.S. and E.I.P.) based on the scoring scale of Rothkopf et al. [29] (Table S4). The final value of the macroscopic assessment of the surgical field was derived from the average of the individual measurements of the assessors. Subsequently, tissue samples were collected from the area of the intervention (including periosteum, epimysium, and subcutaneous tissue) in order to perform a histological examination. The tissue samples were fixed in 10% neutral buffered formalin, processed through graded alcohol and xylene, and embedded in paraffin wax. Paraffin-embedded tissues were sectioned at 4–5 microns and slides were stained with hematoxylin and eosin (H&E) for histopathological examination. During the examination, the following parameters were investigated: the degree of adhesions (areas of necrosis, presence of collagen and blood vessels), the intensity of inflammation (infiltration of histiocytes and lymphocytes), and the quality of healing (fibroblasts/distribution). For the assessment of adhesions, a score ranging from 0, indicating the absence of adhesions, to 3, denoting severe adhesions, was assigned based on the modified scoring system proposed by Yilmaz and colleagues [3] (Table S5). To evaluate the inflammatory response, a score from 0, representing no inflammation, to 3, indicating severe inflammation, was assigned according to the scoring system developed by Yaltirik et al., 2004 [30] (Table S6). Furthermore, a score from 0, representing the worst outcome, to 3, indicating the best outcome, was used for the assessment of healing based on a modified scoring system originally proposed by McMinn [31] (Table S7).

### 2.5. Statistical Analysis

All statistical analyses were performed using Stata 17.0 (Stata Statistical Software, College Station, TX, USA) and the results were interpreted at a 5% level of significance. A mixed-effects linear regression model was employed to investigate the potential association between the applied intervention (in either left or right limbs) and the perimeter of the surgical site. The perimeter was normally distributed (Shapiro–Wilk test *p* = 0.997, W = 0.996). Perimeter was the dependent variable whereas “limb” (either left or right, denoting the corresponding treatment intervention group) and time variable “day” were offered as the independent ones. A random-effect term was incorporated to account for the dependence of observations at the dog level and the longitudinal structure due to repeated measurements in each dog over time.

A mixed-effects ordinal logistic regression model was fit to investigate the potential association of the applied intervention (in either left or right limbs) with the odds of the lameness score. The lameness score was the dependent variable, whereas limb (either left or right, denoting the corresponding intervention) and day were offered as the explanatory variables. A random-effect term was incorporated to account for the dependence of repeated observation within dogs.

Three mixed-effects ordinal logistic regression models were employed to investigate the potential association of the applied intervention (in either left or right limbs) and the findings of ultrasonographic evaluation. Each one of the evaluated parameters, namely the score of “echogenicity and structural organization of the tissue at the fracture site”, the score of “formation of the callus and the vascularization present at the osteotomy”, and the score resulting from the sum of the aforementioned individual scores (denoted as “grade”), were the dependent variables in the equally numbered models; the limb (either left or right, denoting the corresponding intervention) and day were offered as the independent variables. A random-effect term was incorporated to account for the dependence of observations at the dog level and the longitudinal structure design due to repeated measurements in each animal over time, specified in a random-coefficient proportional odds model to allow the slope of time variable “day” to vary randomly between dogs within the left or the right limb. A mixed-effects linear regression model was employed to investigate the potential association between the applied intervention (in either left or right limbs) and the speed of blood flow. The speed of blood flow was the dependent variable, whereas “limb” (either left or right, denoting the corresponding treatment intervention group) and time variable “day” were offered as the independent ones. A random-effect term was incorporated to account for the dependence of observations at the dog level and the longitudinal structure due to repeated measurements in each animal over time.

Regarding radiographic evaluation, our objectives comprised the investigation of potential differences in the radiographic findings between the two treatment groups, especially on D28 post-operatively. Two ordinal logistic regression models were used to investigate the existence of differences in the two groups on D0 and D28. Standards errors were adjusted for clustering of observations within dogs (left and right limbs of the same dog).

Two Wilcoxon signed-rank tests were used to test the null hypothesis of the existence of no difference in the median score of macroscopic evaluation of adhesions between the left and right leg of the tested animals, assigned by each rater. Additionally, a weighted Cohen’s kappa coefficient (κ) for ordinal scale data was calculated to evaluate the agreement between the two raters. Furthermore, the Wilcoxon signed-rank test was used to evaluate the existence of any difference in the median value of each of the histological parameters’ evaluation scores, namely the adhesion score, the inflammation score, and the healing score between the two groups.

## 3. Results

### 3.1. Clinical Examination

Clinical examinations before surgery did not reveal any abnormalities in the animals. Routine examinations performed on all animals during the study did not reveal any clinical signs of infection or dehiscence of the surgical wound, regardless of the group they belonged to.

### 3.2. Perimeter of the Surgical Site

**Figure 1:**
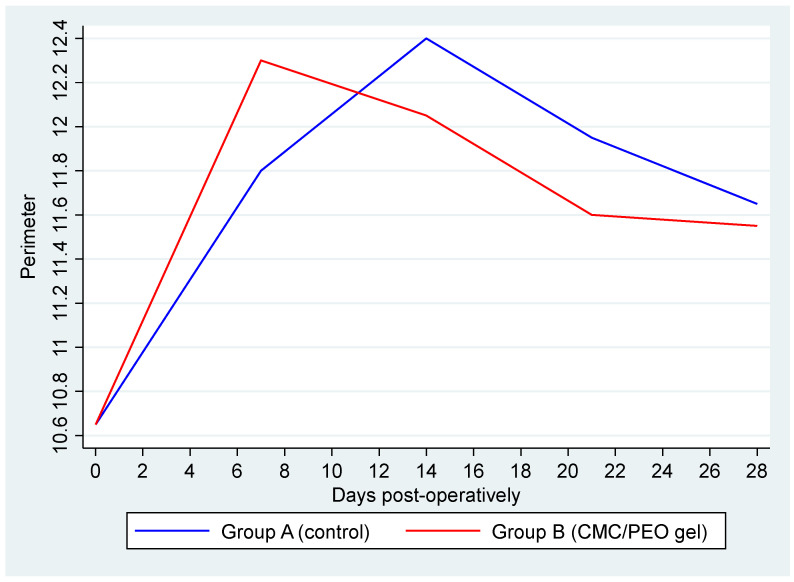
Average perimeter by group over days post-operatively. Group A (control) is represented by the blue line and Group B (CMC/PEO gel) by the red one.

There was no statistically significant difference in the mean value of the perimeter between left and right limbs (*p* = 0.558, Coef. = 0.15, 95% CI: −0.35; 0.65). A statistically significant effect of the time variable “day” was observed (*p* = 0.004); particularly, for one unit increase (i.e., week) of “day”, an increase of 0.03 cm (95% CI: 0.01; 0.051) in the perimeter is expected regardless of the group. The interaction term between the time variable “day” and the intervention applied suggested was not statistically significant (*p* = 0.317) (Table 1 and Figure 1).

### 3.3. Lameness

**Figure 2:**
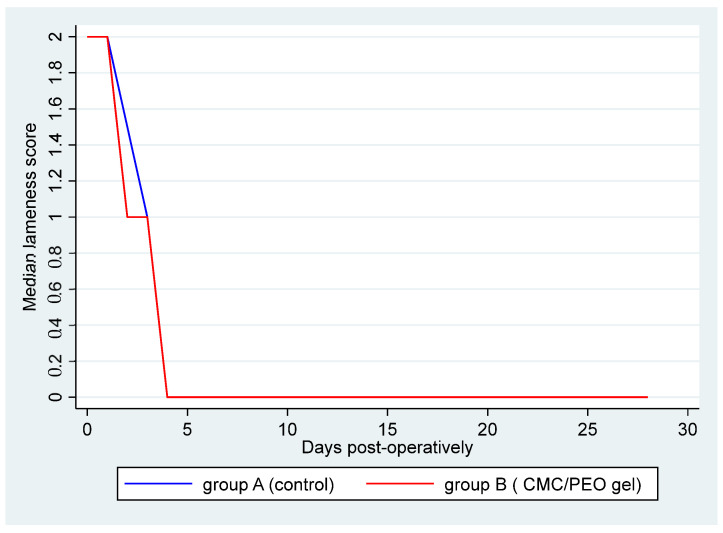
Median lameness score by group over days post-operatively. Group A (control) is represented by the blue line, and group B (CMC/PEO gel) is represented by the red one.

Before the surgery, no abnormalities were detected in the animals during the assessment of lameness. All the animals were assigned a score of 0 on the relevant evaluation scale. During the study period, the lameness score ranged from 0 to 2, with a median value of 0 (IQR: 0–1) (Table 2 and Figure 2). The results of the employed mixed-effects ordinal logistic model suggested that there was no difference in the ordered log–odds of lameness score between group A and group B (*p* = 0.227, OR: 0.33, 95% CI: 0.05; 1.98). Additionally, a statistically significant time effect (*p* < 0.001, OR = 0.0008, 95% CI: 0.00002; 0.032) was observed, suggesting that with each elapsed day post-operatively there was a 0.08% (0.002; 3.2) reduction in the log–odds of a higher versus a lower lameness score, regardless the applied treatment.

### 3.4. Overall Adverse Effects

As evidenced by the results presented above, the use of the CMC/PEO gel was not accompanied by the presence of any adverse events.

### 3.5. Diagnostic Imaging

#### 3.5.1. Ultrasonographic Examination

The distributions of the scores resulting from the ultrasonographic examination, per group, and post-operative day of examination are presented in Table 3.

**Figure 3:**
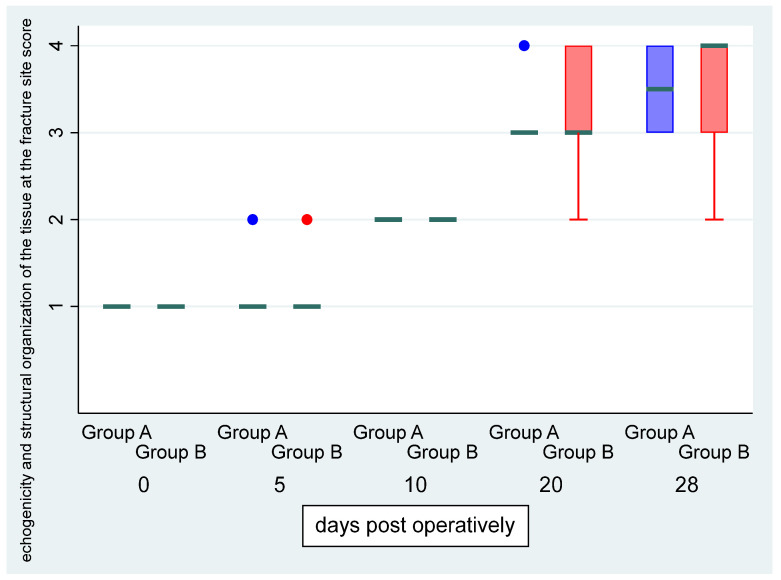
Median (green transverse line) and interquartile range (IQR) of echogenicity and structural organization of the tissue at the fracture site score by group (group A, i.e., control, and group B, i.e., CMC/PEO gel, in blue and red color, respectively) over days post-operatively.

There was no statistically significant difference in the “echogenicity and structural organization of the tissue at the fracture site” score between the left and right limb (OR: 0.77, *p* = 0.643, 95% CI: 0.26; 2.27) (Figure 3). There was a statistically significant effect of the time variable “day” (OR = 1.84, *p* < 0.001, 95% CI:1.43; 2.35), suggesting an expected 1.84 increase in the log–odds of obtaining a higher level of the specific score for one unit increase in “day”.

**Figure 4:**
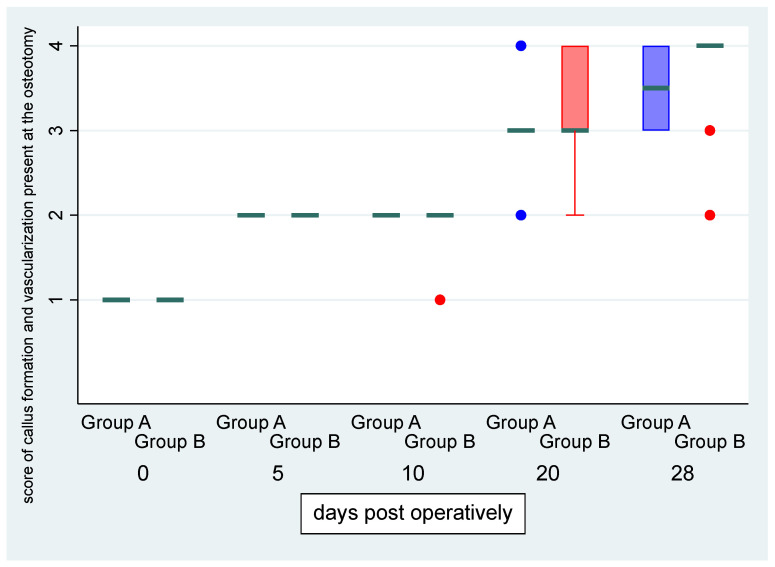
Median (green transverse line) and interquartile range (IQR) of formation of the callus and the vascularization present at the osteotomy score by group (group A, i.e., control, and group B, i.e., CMC/PEO gel, in blue and red color, respectively) over days post-operatively.

There was no statistically significant difference in the “formation of the callus and the vascularization present at the osteotomy” score between the left and right limb (OR: 1.06, *p* = 0.912, 95% CI: 0.36; 3.14) (Figure 4). There was a statistically significant effect of the time variable “day” (OR = 1.80, *p* < 0.001, 95% CI:1.44; 2.244), suggesting an expected 1.8 increase in the log–odds of obtaining a higher level of the above score for one unit increase in “day”.

**Figure 5:**
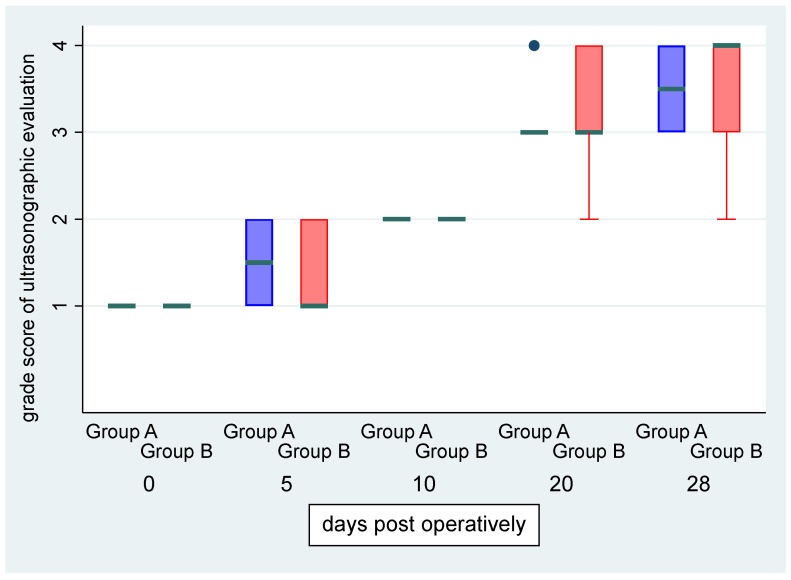
Median (green transverse line) and interquartile range (IQR) of the grade score of ultrasonographic evaluation by group (group A, i.e., control, and group B, i.e., CMC/PEO gel, in blue and red color, respectively) over days post-operatively.

**Figure 6:**
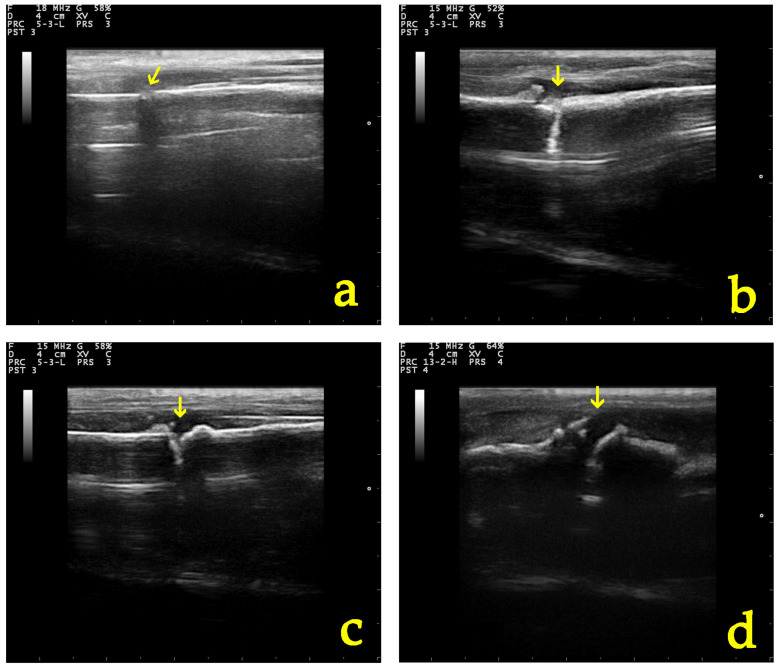
B-mode appearance of the fracture site (yellow arrow) in a group A (control group) dog (**a**) post-operatively, (**b**) 10, (**c**) 20, and (**d**) 28 days after osteotomy.

**Figure 7:**
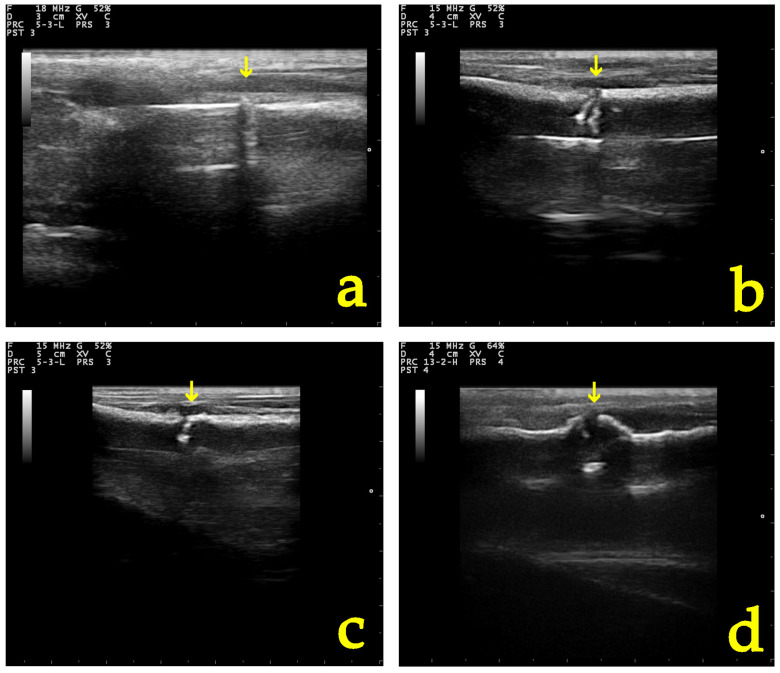
B-mode appearance of the fracture site (yellow arrow) in a group B (CMC/PEO gel) dog (**a**) post-operatively, (**b**) 10, (**c**) 20, and (**d**) 28 days after osteotomy and anti-adhesion agent application.

**Figure 8:**
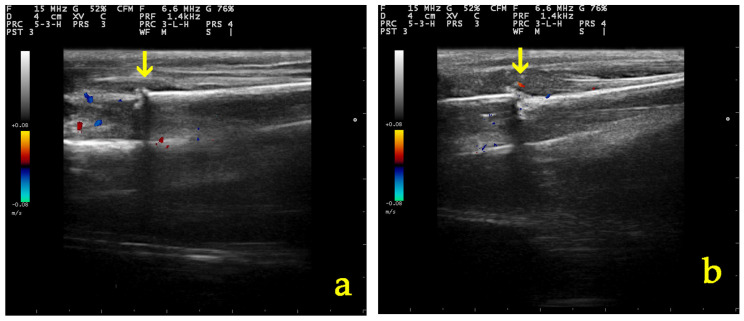
Color Doppler assessment of vascularization in the osteotomy site (yellow arrow) 10 days post-operatively in (**a**) a group A and (**b**) a group B dog.

There was no statistically significant difference in “grade” score between left and right leg (OR: 0.79, *p* = 0.661, 95% CI: 0.27; 2.26) (Figure 5). There was a statistically significant effect of the time variable “day” (OR = 1.79, *p* < 0.001, 95% CI:1.43; 2.24), suggesting an expected 1.79 increase in the log–odds of obtaining a higher score level of “grade” for one unit increase in “day” (Figure 6, Figure 7 and Figure 8).

There was no statistically significant interaction between the time variable “day” and the intervention applied in the left or right leg in all above models (*p* = 0.500, *p* = 0.612 and *p* = 0.956, respectively).

Additionally, for all evaluated parameters through ultrasound examination, fractures having higher scores at day 0 tended to have a greater decline in severity than those with lower scores in both groups.

#### 3.5.2. Radiographic Examination

There was not any statistically significant difference in the radiographic findings between the two groups on day 28 (*p* = 0.379, OR = 1.97, 95% CI: 0.43; 8.89) (Table 4).

### 3.6. Macroscopic and Histological Evaluation

**Figure 9:**
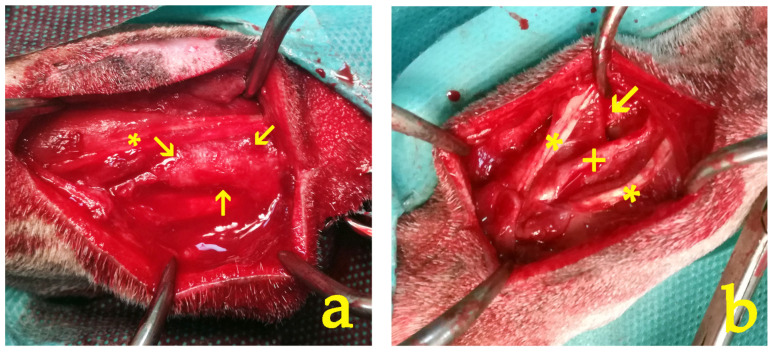
At the 28-day timepoint, the macroscopic appearance of the intervention site revealed distinct differences between the study groups: (**a**) In group A, dense fibrous adhesions (indicated by arrows) were observed in the interspace between the flexor muscle (*) and the ulna bone. These adhesions had to be surgically removed. (**b**) In contrast, group B exhibited fewer, milder, and more filmy adhesions (arrow). These adhesions could be easily eliminated by manual traction alone, without the need for surgical intervention. The ulnar bone (+) and the flexor and ulnar muscles (*) are clearly visible.

There was a statistically significant difference in the median score of macroscopic assessment between the left and right limb for both raters (both *p* = 0.006), whereas the weighted Cohen’s kappa coefficient (κ) suggested a substantial inter-rater agreement (kappa = 0.72, *p* < 0.001) (Figure 9).

**Figure 10:**
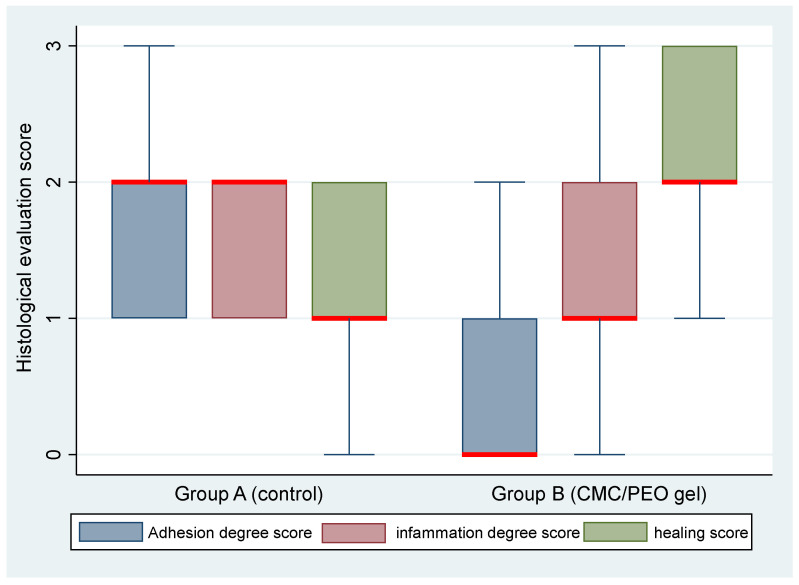
Median (red transverse line) and interquartile range (IQR) of histological evaluation (adhesion degree, inflammation degree, and healing, depicted in blue, maroon, and green color, respectively) scores by group (group A, control, and group B, CMC/PEO gel) on day 28 post-operatively.

**Figure 11:**
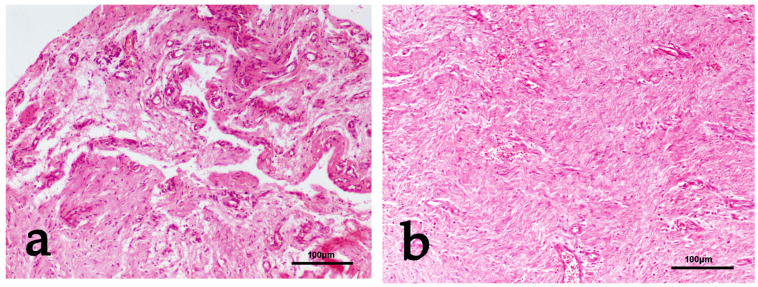
(**a**) Group A: Fine fibrous network of loose connective tissue interrupted by areas of necrosis—tissue depletion mixed with irregular collagen bundles haphazardly distributed. Mild histiocytic and lymphocytic infiltration and increased vasculature are also observed. Adhesions score 3. H&E staining. (**b**) Group B: Dense fibrous connective tissue with wavy arrangement of fibroblasts and regularly distributed vasculature are observed. Adhesions score 0 (absence), quality of healing score 3. H&E staining.

Regarding histopathological examination, there was a statistically significant difference in the median adhesion score (*p* = 0.0049) and the median healing score (*p* = 0.0102) between the two groups. On the contrary, no statistically significant difference in the median inflammation score between the two groups (*p* = 0.5225) was detected (Table 5, Figure 10 and Figure 11).

## 4. Discussion

The development of adhesions during orthopedic trauma can constitute a serious complication in small animals [9,10]. Several studies in humans have shown that adhesions can be significantly reduced through the use of anti-adhesive barriers [15,16,17,18,19,20]. In small animals, such an approach has not been made so far, neither in research nor in clinical practice. Therefore, in order to advance the field of small animal orthopedics and address the issue of adhesions, we considered it necessary to investigate the use of an anti-adhesive barrier in this context. Before it can be used in clinical practice, it was appropriate to investigate the safety of the use of the material and to record any adverse effects. Additionally, it should be clarified that its presence does not affect the normal progression of fracture healing. Crucially, the use of such an anti-adhesive barrier should prevent or substantially reduce the presence of adhesions during the post-operative period of orthopedic procedures, thereby leading to a reduction in morbidity and enabling faster functional recovery.

In the present study, an osteotomy was performed in the middle third of the ulna. This location was chosen for several reasons such as the ease of the surgical approach, the proximity to the flexor and ulnaris muscles of the antebrachium, and, most importantly, the fact that the ulna is not a primary weight-bearing bone of the forelimb; consequently, an osteosynthesis of the osteotomy site was not necessary.

As already mentioned, concerning human orthopedic surgery, various anti-adhesive barriers have been tested both in experimental animal models and in human patients. Several studies have investigated materials such as absorbable oxidized regenerated cellulose or a composite of hyaluronic acid with carboxymethyl cellulose for their potential effect as anti-adhesive barriers, with very promising results [3,16,17,18,20]. However, these fabric barrier materials have exhibited some limitations. For instance, their effectiveness can be reduced in the presence of blood [32]. Additionally, it may be challenging to handle, apply, and cover the entire area of interest during surgery, as the materials tend to lose their integrity and strength upon hydration [20]. For the purposes of this study, we utilized a CMC/PEO gel as the anti-adhesive barrier material. This CMC/PEO formulation is a transparent, viscoelastic gel that can be easily applied to specific anatomical locations where adhesion formation is a potential issue [22]. The effectiveness of this anti-adhesive barrier material may be attributed to the properties of its polymer components, as well as their synergistic effects when combined. The CMC component exhibits tissue-adhesive characteristics, allowing it to form a protective barrier, whereas the PEO component serves to inhibit protein interactions with the CMC, thereby minimizing unwanted tissue adhesion [21,24,25]. Furthermore, the material is absorbed within one month through the process of hydrolysis [25]. In the present study, the CMC/PEO gel proved to be easy to handle and apply, remaining in the intended location due to the tissue-adherent characteristics of the CMC component, in agreement with previous studies [21,23,24]. Furthermore, no adverse effects were recorded.

Inflammation and pain were indirectly assessed during the study period by measuring the perimeter of the surgical site and evaluating the degree of lameness exhibited by the animals. The results showed no significant differences in surgical site perimeter or lameness scores between the two groups. Moreover, both groups demonstrated a statistically notable reduction in lameness throughout the experiment. These findings suggest that the application of the CMC/PEO gel did not cause any additional inflammation or pain at the surgical site. Taken together, this could suggest that the gel was well-tolerated and did not exacerbate the inflammatory response or result in increased discomfort for the animals.

Two clinical studies in humans examined the effect of using an anti-adhesive barrier to minimize adhesion formation after surgical repair of phalangeal fractures. In these studies, fracture healing was also indirectly assessed by estimating parameters such as the range of motion, total angular movement, and pain experienced by the participants. The results of these studies indicated that the use of the anti-adhesive agent did not alter the fracture-healing process [33,34]. However, radiographic imaging continues to be the most widely utilized modality for evaluating fracture healing progression [35]. In the context of this study, a modified radiological scoring system was employed to objectively assess the radiological findings. This scoring system provides a comprehensive assessment by taking into account key bone-related variables, including bone formation, proximal and distal union, and remodeling [28]. The radiographic findings did not reveal any statistically significant differences between the two groups. Notably, both groups exhibited comparable improvement in the healing process by the final day of the experimental period. These findings are in agreement with the results of another study conducted in a rabbit model, where the effect of an anti-adhesive barrier on bone healing was investigated. In that study, the radiographic findings obtained four weeks after surgery did not reveal any statistically significant differences between the control group and the group that received the anti-adhesive barrier [36]. In our study the relatively low radiographic assessment scores and, perhaps, the lack of significant differences observed between the groups during the experimental period were not entirely unexpected. This is because radiography is a less sensitive modality for evaluating the early stages of bone formation, particularly the soft callus formation [37]. Given that the study period was relatively short, lasting only 28 days, the radiographic findings may not have been able to adequately capture the subtle changes in the bone healing process. A more sensitive assessment technique may have been required to detect any potential differences in the early stages of fracture healing between the groups during this relatively brief experimental period. Ultrasonography can serve as a more accurate method for detecting the early stages of callus formation and monitoring its progression towards the development of bridging new bone [37,38,39,40,41]. The use of B-mode ultrasonography facilitated the evaluation of the structural organization of the tissue at the fracture site and assessed the potential of the bone healing process. Based on the results of the present study, the echogenicity and structural organization of the tissue at the fracture site, as well as the formation of the callus present at the osteotomy site, did not differ significantly between the two groups. Both groups exhibited a statistically significant effect over time, suggesting that the process of fracture healing was progressing normally in all animals. Moreover, the early stages of bone healing are critically dependent on blood perfusion at the fracture site and the surrounding soft tissues, as this provides the necessary oxygen and nutrients to support the healing process. Previous studies in both animal models and humans have highlighted the utility of Color or Power Doppler imaging for the time-dependent evaluation of the development and regression of vascularization during fracture healing of bones [27,37,42,43,44,45]. In the present study, the vessel development and density within the osteotomy site remained within normal ranges in both groups throughout the study period. This suggests that the CMC/PEO gel, used to prevent adhesion formation was able to create a favorable environment that supported vascularization and blood flow at the fracture site.

The macroscopic evaluation revealed significantly fewer adhesion formations in group B, in which the CMC/PEO gel was applied. These adhesions could be easily eliminated with manual traction. These macroscopic findings were corroborated by the results of the histological examination, which in the CMC/PEO group most often showed fibrous connective tissue with a wavy arrangement of fibroblasts and a regularly distributed vasculature. In contrast, animals in group A exhibited a markedly different histological profile, characterized by a fibrous network of loose connective tissue interrupted by areas of necrosis and tissue depletion, mixed with irregularly distributed collagen bundles. Additionally, according to the results from the histological evaluation, the use of the CMC/PEO gel appeared to enhance soft tissue healing in comparison to the results observed in the control group. The findings from the present study align with the results of a similar investigation conducted in a rat model. In that previous study, the use of the CMC/PEO compound was found to result in significantly lower levels of peritendinous adhesions, as observed through both macroscopic and histological examination [19]. These findings suggest that the use of the CMC/PEO gel was significantly more effective in reducing adhesion formation compared to the control group. Overall, the above findings suggest that the use of the CMC/PEO gel was significantly more effective in reducing adhesion formation and enhancing tissue healing compared to the control group.

## 5. Conclusions

This is the first study to evaluate the efficacy of an adhesion barrier in orthopedic procedures performed in dogs. According to the findings of the study, the application of the CMC/PEO gel was well-tolerated, without any observed adverse effects. Furthermore, its use did not result in additional inflammation or pain at the surgical site compared to the control group. Radiographic and ultrasound evaluations showed no significant differences in fracture healing progression between the two groups, indicating that the CMC/PEO gel did not impair the normal bone healing process. The macroscopic and histological examination findings were even more compelling, revealing significantly fewer and less severe adhesions in the group where the CMC/PEO gel was applied, supporting the potential clinical application of this anti-adhesive barrier in small animal orthopedic procedures.

## Figures and Tables

**Table 1:** Average values and standard deviation (SD) of perimeter (in cm) per treatment group over post-operative days of evaluation.

	Group	D0	D7	D14	D21	D28
Average perimeter (cm) (SD)	A	10.65 (1.00)	11.8 (0.95)	12.4 (0.77)	11.95 (0.86)	11.65 (0.88)
	B	10.65 (1.00)	12.3 (0.95)	12.05 (0.79)	11.6 (0.99)	11.55 (0.79)

**Table 2:** Median values and range of lameness per treatment group over post-operative days of evaluation.

Group	D–1	D1	D2	D3	D4	D5	D6	D7	D14	D21	D28
A	0	2 (1–2)	1.5 (1–2)	1 (0–1)	0 (0–1)	0 (0–0)	0 (0–0)	0 (0–0)	0 (0–0)	0 (0–0)	0 (0–0)
B	0	2 (1–2)	1 (1–2)	1 (0–1)	0 (0–0)	0 (0–0)	0 (0–0)	0 (0–0)	0 (0–0)	0 (0–0)	0 (0–0)

**Table 3:** Median values and range of the examined ultrasonographic parameters score per treatment group over days post-operatively.

Parameter of Ultrasonographic Examination	Group	D0	D5	D10	D20	D28
Echogenicity and structural organization of the tissue at the fracture site	A	1 (1–1)	2 (1–2)	2 (2–2)	3 (3–4)	3.5 (3–4)
	B	1 (1–1)	1 (1–2)	2 (2–2)	3 (2–4)	4 (2–4)
Formation of the callus and the vascularization present at the osteotomy	A	1 (1–1)	2 (2–2)	2 (2–2)	3 (2–4)	3.5 (3–4)
	B	1 (1–1)	2 (2–2)	2 (1–2)	3 (2–4)	4 (2–4)
Total grade	A	1 (1–1)	1.5 (1–2)	2 (2–2)	3 (3–4)	3.5 (3–4)
	B	1 (1–1)	1 (1–2)	2 (2–2)	3 (2–4)	4 (2–4)

**Table 4:** Median values and range of the radiographic evaluation score per treatment group over days post-operatively.

	Group	D0	D28
Radiographic evaluation score	A	0 (0–0)	2 (1–3)
	B	0 (0–0)	3 (1–3)

**Table 5:** Median values and range of the macroscopic and histological assessment scores per treatment group on day 28 post-operatively.

Parameters of Macroscopic and Histological Assessment		Group	D28
Macroscopic assessment	Median adhesion score (range)	A	3 (2–3)
		B	1 (0–2)
Histological examination	Median adhesion score (range)	A	2 (1–3)
		B	0 (0–2)
	Median inflammation score (range)	A	2 (1–2)
		B	1 (0–3)
	Median healing score (range)	A	1 (0–2)
		B	2 (1–3)

## Data Availability

The data presented in this study are available in the article and Supplementary Materials.

## Supplementary Materials

The following supporting information can be downloaded at: https://www.mdpi.com/article/10.3390/vetsci11080343/s1, Table S1: Scoring system for assessing lameness; Table S2: Scoring system for the ultrasonographic assessment of fracture healing; Table S3: Scoring system for the radiographic evaluation of bone healing; Table S4: Scoring system for the macroscopic evaluation of adhesions; Table S5: Scoring system for the histological assessment of adhesions; Table S6: Scoring system for the histological assessment of the inflammatory response; Table S7: Scoring system for the histological assessment of tissue healing, Figure S1: Surgical procedure in the right limb (group B).
