# Supplementary material for: Prevention of Adhesions after Bone Fracture Using a Carboxymethylcellulose and Polyethylene Oxide Composite Gel in Dogs
Source: Vet Sci. 2024 Jul 29;11(8):343. doi: 10.3390/vetsci11080343 (PMC11360353; doi:10.3390/vetsci11080343)

Prevention of Adhesions after Fracture using a Carboxymethylcellulose and Polyethylene Oxide Composite Gel in Dogs

Aikaterini I. Sideri, Elena I. Pappa, Vassilis Skampardonis, Mariana Barbagianni, Stefanos G. Georgiou, Dimitra Psalla, Christina Marouda, Nikitas N. Prassinos, Apostolos D Galatos, Pagona G Gouletsou

Table S1: Presentation of the standardized system for assessing lameness at a walk and trot in dogs (Witte, P.; Scott, H.. Investigation of lameness in dogs. *In Practice*. **2011**, vol 33, 58–66)

Grade	Description
0 (None)	No lameness is observed at a walk or trot
1 (Mild)	Lameness is present, but may only be consistently apparent at a trot
2 (Mild to moderate)	Mild lameness is obviously present at a walk and is worse at a trot
3 (Moderate)	Obvious lameness is present at both gaits
4 (Moderate to severe)	Obvious lameness is present at both gaits and may be intermittently non-weight-bearing
5 (Severe)	Lameness is non-weight-bearing most or all of the time

Table S2: Presentation of the assessment used to monitor the stages of fracture healing based on characteristics of B-mode ultrasonographic images (Pozzi, A.; Risselada M.; Winter M M. Assessment of fracture healing after minimally invasive plate osteosynthesis or open reduction and internal fixation of coexisting radius and ulna fractures in dogs via ultrasonography and radiography *J Am Vet Med Assoc* 2012,241,744–753.

Stage of fracture healing	Characteristics
1	Tissue within the fracture gap appears anechoic to hypoechoic, with possible hematomas and fragments; no vascularization is visible by use of power Doppler ultrasonography.
2	Tissue appears hypoechoic (soft tissue callus) but is now heterogeneous; vascularization (determined by the use of power Doppler ultrasonography) is clearly present in the soft tissue.
3	Evidence of bridging of the fracture gap with inhomogeneous tissue (mix of hypoechoic and hyperechoic areas); vascularization (determined by use of power Doppler ultrasonography) is present but less abundant than in stage 2.
4	Increasingly homogeneous, hyperechoic image of the tissue at the fracture site (acoustic shadow returns); vascularization is still present and appears to be located on the bone surface rather than in the soft tissue.
5	Mature callus is present; homogeneous, hyperechoic tissue bridging the facture gap; the surface of this bridge will progressively appear smoother, compared with previous interrogations; vascularization is not detectable by use of power Doppler ultrasonography.

Table S3. Presentation of the scoring system utilized for the radiographic evaluation of bone formation, union, and remodeling processes [Lane, J.M. and Sandhu, H. Current Approaches to experimental bone grafting. *Orthop. Clin. North Am.* **1987**, *18*, 213–225.].

Score	Description of findings
	Bone formation
0	No evidence of bone formation
1	Bone formation occupying 25% of the defect
2	Bone formation occupying 50% of the defect
3	Bone formation occupying 75% of the defect
4	Bone formation occupying 100% of the defect
	Union (proximal and distal evaluated separately)
0	No union
1	Possible union
2	Radiographic union
	Remodeling
0	No evidence of remodeling
1	Remodeling of medullary canal
2	Full remodeling of cortex

Table S4. Presentation of the modified scoring system utilized for the macroscopic evaluation of adhesions.
(Rothkopf DM, An experimental model for the study of canine flexor tendon adhesions, *J Hand Surg*, **1992**,16A, 694-700)

Score	Adhesion observed
0	No adhesions
1	Few, mild, filmy adhesions that can be eliminated by minimal manual traction
2	Moderate adhesion that can be eliminated by manual traction
3	Many adhesions that cannot be easily eliminated by manual traction
4	Dense fibrous adhesions that must be surgically removed

Table S5. Histological assessment of adhesions was assigned based on the modified scoring system proposed by Yilmaz and colleagues (Yilmaz E. et al. The effect of seprafilm on adhesion formation and tendon healing after flexor tendon repair in chicken. Orthopedics. 2010;33,164-70).

Adhesion	Findings	Score
No adhesion	Normal	0
Light degree adhesion (good)	Few fibers.; fine, long fibers structure	1
Moderate adhesion	Average number of fibers; large, thick fibers structure	2
Severe adhesion	Loss of fibers structure; dense fibrosis	3

Table S6. Histological assessment of the inflammatory response according to the scoring system developed by and colleagues (Yaltirik M., et al. Reactions of connective tissue to mineral trioxide aggregate and amalgam. *J Endod.* **2004**,30,95-9).

Grade of inflammation	Inflammatory response
Grade 0	Absence of inflammatory cells
Grade 1	Mild inflammation, an average of fewer than 25 inflammatory cells per high-power field
Grade 2	Moderate inflammation, an average of 25-124 inflammatory cells per high-power field
Grade 3	Severe inflammation, an average of 125 or more inflammatory cells per high-power field

Table S7. Histological assessment of healing based on a modified scoring system originally proposed by McMinn (McMinn R. Skin and subcutaneous tissues. Vol. tissue repair. New York and London: Academic Press; 1969. 1–40).

Grade	Fibroblasts and distribution
0	No evidence
1	Present only in the perivascular spaces, around new capillaries
2	Present in <50% of the wound tissue examined
3	Present in >50% of the wound tissue examined

Figure S1. Surgical procedure in the right limb (group B). (*α*) The subcutaneous fat and superficial antebrachial fascia were incised between the tendons of the ulnaris lateralis and the lateral digital extensor muscle. (b) Retraction of the tendons and fascia exposed the middle third of the ulna diaphysis. (c) A complete cross-section osteotomy was performed (arrow). (d) One layer of CMC/PEO gel (Oxiplex; FzioMed, Inc., CA, USA) was applied in the space between the osteotomy area and the adjacent muscles.

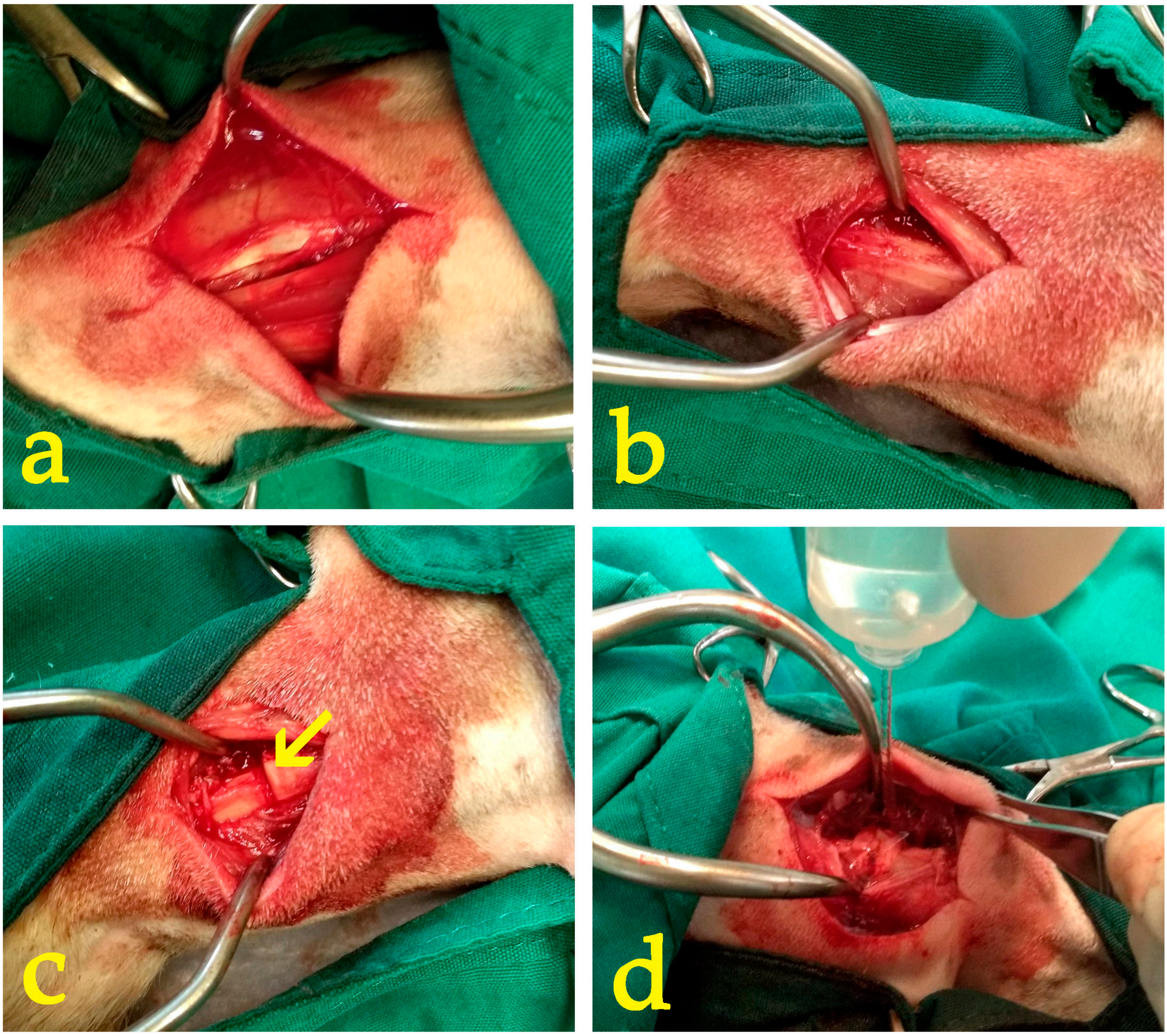

Supplement: Supplementary file 1 [file vetsci-11-00343-s001.zip › vetsci-3080106-supplementary.pdf]
